# Selection of a MCF-7 Breast Cancer Cell Subpopulation with High Sensitivity to IL-1**β**: Characterization of and Correlation between Morphological and Molecular Changes Leading to Increased Invasiveness

**DOI:** 10.1155/2012/609148

**Published:** 2012-05-10

**Authors:** Eloy Andres Pérez-Yépez, Jorge-Tonatiuh Ayala-Sumuano, Alicia Maria Reveles-Espinoza, Isaura Meza

**Affiliations:** ^1^Department of Molecular Biomedicine, Center for Research and Advanced Studies of the National Polytechnic Institute, 2508 Instituto Politecnico Nacional Avenue, 07360 Mexico City, Mexico; ^2^Department of Cell Biology, Center for Research and Advanced Studies of the National Polytechnic Institute, 2508 Instituto Politecnico Nacional Avenue, 07360 Mexico City, Mexico

## Abstract

Cancer and inflammation are closely related in tumor malignancy prognosis. Breast cancer MCF-7 cells have a poor invasive phenotype, although, under IL-1**β** stimulus, acquire invasive features. Cell response heterogeneity has precluded precise evaluation of the malignant transition. MCF-7A3 cells were selected for high sensitivity to IL-1**β** stimulus, uniform expression of CXCR4, and stability of IL1-RI. Structural changes, colony formation ability, proliferation rate, chemotaxis, Matrigel invasion, E-cadherin mRNA expression and protein localization were determined in these cells and in MCF-7 parental cells under the stimulus of IL-1**β**. Selected MCF-7A3 cells showed a uniform response to IL-1**β** stimulation increasing features of invasive cells such as scattering, colony formation, proliferation, chemokinesis and invasion. Basal expression of E-cadherin mRNA was higher, and IL-1**β** stimulus had no further effect at early times of cytokine exposure. Total E-cadherin levels remained unchanged in parental cells, whereas levels decreased, as MCF-7A3 cells became fibroblastoid or scattered. Triton X-100 soluble/insoluble E-cadherin ratios were highly increased in these cells, while, in MCF-7pl cells, ratios could not be correlated with morphology changes. MCF-7A3 cells uniform response to IL-1**β** allowed characterization of changes induced by the cytokine that had not been assessed when using heterogeneous cell lines.

## 1. Introduction

Breast cancer represents an important worldwide problem of public health with more than 1.2 million reported cases and a mortality of 400,000 patients each year. Research on this topic, mainly carried out in *in vitro* models, has shown that breast epithelial cells are susceptible to undergo deregulation of the cell cycle under the effect of environmental stimuli. This deregulation leads to cell proliferation and development of a tumor. Progression to cancer malignancy occurs when transformed cells migrate to other tissues and develop secondary tumors. In this process of metastasis, cancer cells undergo an epithelial-mesenchymal transition (EMT) that allows cell migration through tissues and blood vessels. At the onset of EMT, cell-cell or cell-stroma junctions of the cancer cells are disrupted followed by delocalization of molecular components of the junctions. Delocalization of epithelial protein markers such as E-cadherin, desmoplakin, and ZO-1 leads to their degradation, whereas a gradual increase of mesenchymal markers such as N-cadherin, vimentin, and nuclear *β*-catenin occurs [1]. These changes in molecular markers have been related to the level of malignancy of cancer cells [2] and a nonfavorable prognosis in patients [3, 4]. Chronic inflammation in the tumor microenvironment is considered a factor involved in development of malignancy of several types of cancer; also, proinflammatory cytokines have been proposed as important inductors of carcinogenesis, invasiveness and metastasis [5]. Interleukin 1*β* (IL-1*β*) is known to be present in mammary tumors, and its higher expression in the tumor microenvironment suggests its possible role in metastasis [6, 7]. Recent *in vitro *work published by our group has demonstrated that the poorly invasive MCF-7 cell line of mammary epithelial cancer acquires several features of an invasive phenotype under the stimulus of IL-1*β* [8, 9]. Under these conditions, major structural changes such as loss of cell-cell contact, acquisition of a fibroblastoid cytoarchitecture and cell scattering were observed. Additionally, increased cell migration and invasion of extracellular protein matrices occurred concomitantly with higher expression of the CXCL12 chemokine receptor, CXCR4. Nonetheless, significant decrease of E-cadherin levels in the IL-1*β* stimulated cells was not detected [8, 9]. In contrast, a previous report has shown that invasiveness and metastasis correlated with delocalization and loss of this epithelial cell protein marker *in vivo *and *in vitro *models [10]. Further studies using breast and peritoneal cancer cells have shown that E-cadherin loss, induced by IL-*β*, required certain conditions such as higher concentration of glucose in the culture medium or keeping the cells in subconfluent conditions [11–13]. Recently, it has been reported that arachidonic acid promotes an EMT-like transition in nontumorigenic mammary MCF-10A cells without eliciting changes in E-cadherin expression [14] and invasive mammary cancer cells (MDA-MB-435) transfected to overexpress E-cadherin retain their invasive phenotype [15]. The above differences in the response of mammary cells to IL-1*β* stimulus might be due to the known heterogeneity and instability of cancer cell lines that could hinder interpretation of results when employing these cells. Considering this problem, we selected an MCF-7 cell subpopulation with increased sensitivity to IL-1*β* stimulus and uniform expression of the CXCR4 receptor, a molecule known to be a factor in cell migration and malignancy. Our results showed that more than 90% of this subpopulation responded to the cytokine stimulus with uniform, programmed changes of cell shape, scattering, proliferation, chemokinesis and invasiveness, concomitantly with sequential delocalization of E-cadherin from the cell membrane, its accumulation in the cytoplasm, and its degradation. Upregulation of E-cadherin mRNA expression in nonselected MCF-7 cells (MCF-7pl) by IL-1*β* was correlated with early times of stimulation when E-cadherin in some cells was beginning to separate from the membrane into the cytoplasm. However, as in these cells, the response to the stimulus was heterogeneous, the initial increase of E-cadherin mRNA levels was followed by a decrease, while protein levels remained without variation. In contrast, in MCF-7A3 cells, E-cadherin mRNA expression decreased at earlier times and decrease of the protein was only noticeable around 72 h. At later times, when most cells, were scattered, E-cadherin mRNA levels significantly dropped and very low levels of E-cadherin were detected.

Although tumors are comprised by distinct subpopulations that elicit a specific phenotype, our results showed that selected cancer cell subpopulations, uniformly responsive to specific stimuli, could be reliable strategy to study specific signaling pathways and differential stages induced by IL-1*β* during a phenotypic transition to malignancy.

## 2. Results

### 2.1. Selection of the MCF-7 Cell Subpopulation Uniformly Responsive to IL-1*β* Stimulus

**Figure 1:**
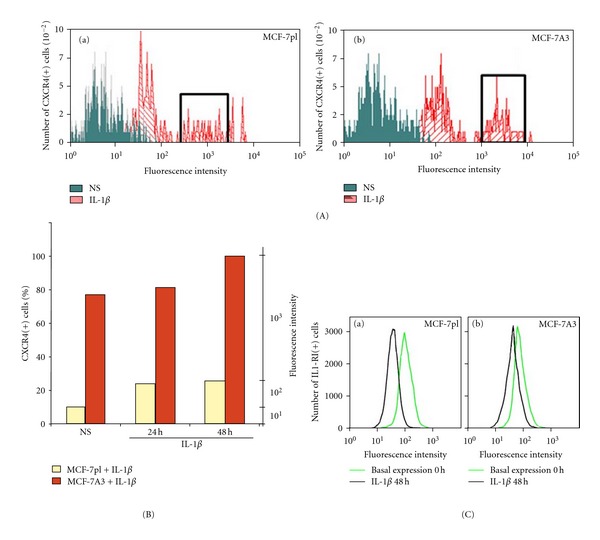
Isolation of subpopulation MCF-7A3 by selection of CXCR4 and IL1-RI expression. MCF-7 cells were starved for 18 h in culture media containing 0.1% FBS then stimulated with IL-1*β*. After 48 h, cells were labeled with anti-CXCR4 antibody or with anti-IL1-RI antibody and analyzed by flow cytometry. (A) Higher expression of CXCR4 by cell subpopulations as selection marker. IL-1*β*-stimulated cells were selected by three rounds of sorting. Boxes indicate the subpopulation that was isolated in the first and last rounds. (B) Percentage of cells expressing CXCR4 in MCF-7pl and MCF-7A3 cells nonstimulates and IL-1*β* at 24 and 48 h. (C) Expression of receptor IL1-RI in MCF-7pl and MCF-7A3 cells in basal conditions and under the stimulus of IL-1*β*.

High expression of the CXCR4 receptor of chemokine CXCL12 in cancer cell lines has been related to invasiveness, malignancy, and homing [16]. Poorly invasive mammary epithelial cancer MCF-7 cells are not metastatic, although they express basal levels of CXCR4 [17]. It has been shown that *in vitro* exposure of these cells to proinflammatory cytokine IL-1*β* induces higher expression of CXCR4 closely followed by striking changes of cell morphology and behavior patterns that resembled those expected in cells going through a transition to an invasive phenotype [8, 9]. However, these changes were not homogeneous as only a fraction of the cell population responded to the cytokine stimulus. This heterogeneity, widely observed in cultured cancer cell lines, has impaired interpretation of results, leading to controversy [18]. Searching for an appropriate way to surmount this problem, a subpopulation of MCF-7 cells was obtained after selection for a higher and uniform expression of CXCR4 after stimulation with IL-1*β*. For this, parental MCF-7 cells (MCF-7pl) were stimulated with the cytokine for 48 h and sorted after being labeled with fluorescent anti-CXCR4 antibody. The first round of selection showed that 26% of the cells increased in one order of magnitude the expression of CXCR4 above the expression showed by nonstimulated cells (Figure 1(A(a)), box). The cells expressing higher levels of CXCR4 were recovered and subjected to two additional rounds of selection. The final selected cells, MCF-7A3 (Figure 1(A(b)), box), composed 43% of the population and showed an increase of CXCR4 expression closer to two orders of magnitude above the expression observed in MCF-7pl cells. Parental and selected cells were then cultured in the same conditions to prepare banks of the same cell passage and be used in all the experiments reported here. In the absence of IL-1*β*, 10% of MCF-7pl cells expressed low baseline levels of CXCR4, while 77% of MCF-7A3 cells showed expression of the receptor two orders of magnitude higher (Figure 1(B)). Exposure of the cells to IL-1*β* for 24 h increased to 82% the MCF-7A3 cells expressing higher levels of the receptor, but only 24% of MCF-7pl cells did. At 48 h, almost 100% of the MCF-7A3 cells responded to the cytokine stimulus, whereas the percentage of responding MCF-7pl cells remained at 26%. At this time, stimulated MCF-7A3 cells expressed CXCR4 levels with values above one order of magnitude higher than stimulated MCF-7pl cells. These results showed that the selected MCF-7A3 cell subpopulation expressed not only higher baseline levels of CXCR4 than nonselected cells, but that selected cells responded uniformly to IL-1*β* by expressing higher levels of the receptor on the cell surface.

### 2.2. Expression of the IL-1*β* Receptor in MCF-7A3 Cells

The receptor IL-1RI for IL-1*β* is expressed in many types of eukaryotic cells. Binding of IL-1*β* to the extracellular domain of this receptor activates several signaling pathways [19, 20]. Our previous studies with nonselected MCF-7 cells demonstrated that IL-1*β* activates signaling pathways that induced striking rearrangements of the actin cytoskeleton followed by changes of cell shape and increased motility [8]. These results and the observed increased expression of CXCR4 in these cells, also induced by their exposure to IL-1*β* [9], suggested that IL-1RI expression could be modulated by IL-1*β*, as reported in human granular lymphocytes [21]. Thus, we evaluated expression of IL-1RI in MCF-7pl and MCF-7A3 cells, under nonstimulated and IL-1*β*-stimulated conditions. Cells were labeled with anti-IL1-RI antibody tagged with FITC. Fluorescence intensity and the number of positive cells were determined by flow cytometry. As shown in Figures 1(C(a)) and 1(C(b)), MCF-7pl and MCF-7A3 cells have similar basal expression of IL-1RI on the cell surface under nonstimulated conditions. However, when cells were stimulated with IL-1*β* for 48 h, MCF-7pl cells lost receptor from their surface as indicated by antibody fluorescence decrease in almost one order of magnitude, compared to MCF-7A3 cells. When analyzing the mean fluorescence intensity corresponding to IL1-RI present on the cell surface, it was decreased about 9-fold in MCF-7pl cells, while in MCF-7A3 cells, only decreased 1.19-fold. These results suggest that IL-1*β* may upregulate the expression of the receptor or induce its faster recycling in MCF-7A3 cells. 

### 2.3. Enhanced Cell Scattering and Colony Formation of MCF-7A3 Cells

**Figure 2:**
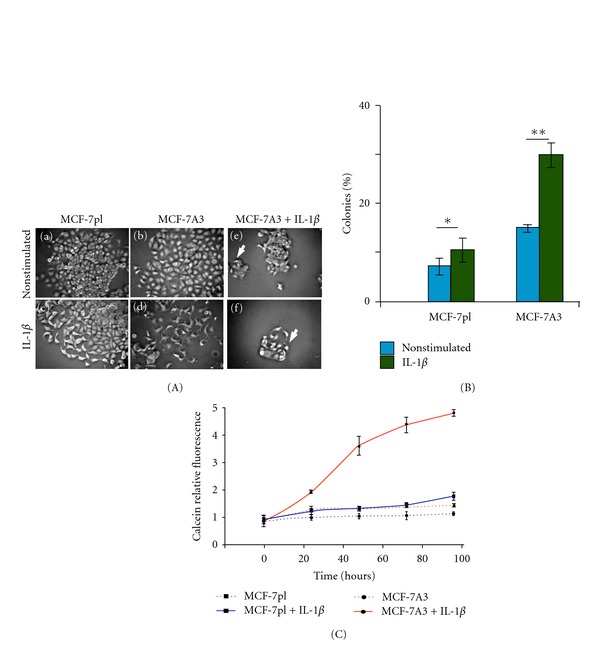
MCF-7A3 cells show increased sensitivity to IL-1*β* demonstrated by their higher scattering, formation of colonies, and cell proliferation. Cells were seeded at very low density (200 cells per 100 mm dish) and allowed to grow until well-defined individual colonies were formed. Cells were then stimulated with IL-1*β* for 48 h. (A) Representative phase-contrast micrographs of colonies of MCF-7pl and MCF-7A3 cells show the difference in cell scattering under IL-1*β* stimulation. Arrowheads point to representative satellite colonies formed in MCF-7A3 cells after IL-1*β* stimulus. (B) Quantification of the colonies formed by the two subpopulations in nonstimulated and stimulated conditions. Data are expressed as mean ± SD of triplicated experiments. *P = 0.03; **P = 0.005. Comparisons were made between results from nonstimulated and IL-1*β*-stimulated conditions in the same cell subpopulation (Mann-Whitney *U* test). (C) Proliferation rate of MCF-7 and MCF-7A3 cells expressed as increase of calcein-AM fluorescence. Cultures of both cell types were stimulated with IL-1*β* as previously indicated. To determine proliferation rate, calcein-AM was added to cultures at 0, 24, 48, 72, and 96 h for 2 h. Calcein fluorescence emitted only by live cells was measured at 485 nm for excitation and 520 nm for emission. Values are presented as relative fluorescence related to time 0 h and it is the mean ± SD of three independent experiments.

Having proved the different sensitivity of MCF-7A3 and MCF-7pl cells to IL-1*β* stimulus, the capacity of these cells to undergo changes in their epithelial morphology and scatter from formed colonies, as expected from cells going through a phenotypic transition, was analyzed in nonstimulated and IL-1*β*-stimulated cells for 48 h. As shown in Figure 2(A(a)), MCF-7pl cells grew forming compact colonies with typical epithelial polygonal shape in close contact with each other. Few cells, in the border of the colonies, showed fibroblastoid shape and had moved away from the colony mass. In contrast, colonies formed by MCF-7A3 cells (Figure 2(A(b))) were not compact, they did not show tight contacts with neighboring cells, and many of them had moved away from the parental colony, alone or in groups. When MCF-7pl cells were stimulated with IL-1*β* for 48 h, more fibroblastoid cells were seen scattered from the border of the colonies and showing cytoplasmic projections suggestive of a migratory phenotype (Figure 2(A(c))), as previously reported by our group [8]. These structural changes were clearly evident in the great majority of MCF-7A3 cells stimulated with IL-1*β* (Figure 2(B(d))). In addition, in these cultures, numerous small satellite colonies close to larger colonies were observed (Figure 2(A(e)), arrow). In few of the remaining colonies, cells were seen in the process of detaching from each other (Figure 2(A(f)), arrow). These results showed that the selected subpopulation responded uniformly to IL-1*β* induction of structural changes. 

The observed differences between the colonies formed by the two types of cells suggested a possible relation with the higher capacity of MCF-7A3 cells to respond to IL-1*β*. Thus, as a first approach, we assessed the ability of these cells to form colonies. MCF-7pl and MCF-7A3 cells were seeded at very low density (200 cells/100-mm dish) and cultured to obtain well-defined colonies. The number of colonies in each dish was counted before and after IL-1*β* stimulation. In absence of the cytokine, MCF-7A3 cells showed a higher than 2-fold increase of the number of colonies formed in respect to those formed by MCF-7pl cells (Figure 2(B)). After stimulation with IL-1*β* for 48 h, MCF-7A3 cells showed a further increase to 3-fold compared to the parental cells (Figure 2(B)) due to the increased number of satellite colonies formed in these cultures (Figures 2(A(e)) and 2(A(f))). Quantification of the number of colonies in MCF-7A3 cells, containing dispersed cells after stimulus with IL-1*β*, showed that 96% of them contained this type of cells, while MCF-7pl cultures only contained 56%. These and the above data showed a clear increase in the number of MCF-7A3 cells that proliferated and moved away from initially formed colonies after IL-1*β* stimulation compared to MCF-7pl cells and suggested that MCF-7A3 cells uniformly activate migration and proliferation mechanisms, while nonselected cells responded heterogeneously to IL-1*β*.

### 2.4. Higher Proliferation Rate of MCF-7A3 Cells

To test if IL-1*β* stimulus increased cell proliferation, subconfluent cultures of MCF-7pl and MCF-7A3 cells (5000 cell/60 mm plate) were stimulated with IL-1*β* and cultured up to 96 h. At 24, 48, 72, and 96 h, calcein-AM was added with fresh medium for 2 h. Parallel cultures not exposed to IL-1*β* were maintained under the same conditions and also incubated with the fluorochrome. The fluorescence emitted by calcein (which is only processed by live cells) was quantified in each condition by fluorometry and the values obtained normalized to values at time zero and interpolated in a control curve prepared to determine the number of cells corresponding to established fluorescence values. Figure 2(C) shows that proliferation rate in MCF-7pl cells slowly increased to reach a value 2-fold higher at 48 h when stimulated with IL-1*β*. In contrast, the cytokine stimulus increased proliferation rate of MCF-7A3 cells up to 3.5-fold at the same time (Figure 2(C)). Similar results were obtained when cells cultured in the same conditions were counted in Neubauer chambers (not shown). These results showed that, although IL-1*β* stimulus induced a significant increase in the proliferation rate of MCF-7pl cells, in MCF-7A3 cells, the induced increase was more than 3.5-fold higher.

### 2.5. MCF-7A3 Cells Increased Chemotaxis and Invasiveness

**Figure 3:**
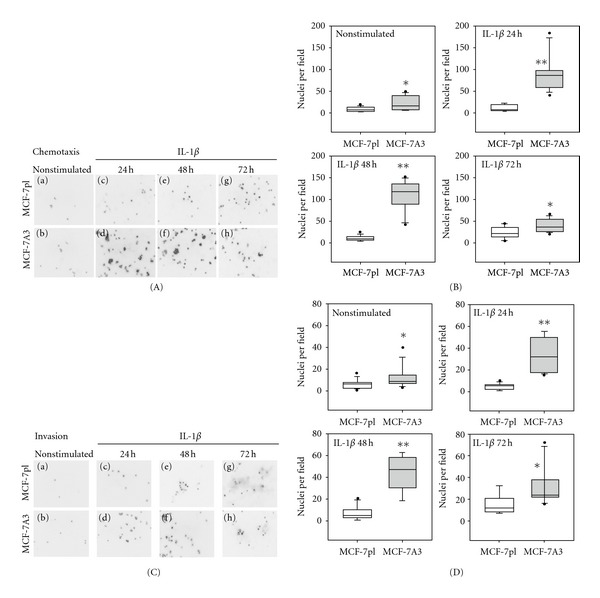
MCF-7A3 cells showed increased chemotaxis and invasiveness than parental MCF-7pl cells. (A) Chemotaxis assay. MCF-7 and MCF-7 A3 cells were stimulated with IL-1*β* for 24, 48, 72 h. After stimulus, cells were transferred to Transwell chambers and allowed to migrate for 24 h in presence of the chemoattractant, CXCL12. Cells that migrated to the lower side of the filter were fixed with PFA and nuclei stained with DAPI. Fluorescent nuclei were counted in acquired digital images of 10 randomly chosen fields in each condition. (B) Box plots show the number of cells that migrated after IL-1*β* stimulus. (C) Invasion assay. Cells were treated in a similar way as in the chemotaxis assay, except for the use of a Matrigel layer deposited on top of the filter of the chamber and the absence of the chemoattractant CXCL12 in the culture medium. (D) Box plots show the number of cells that invaded and broke down Matrigel to cross to the lower side of the filter after IL-1*β* stimulus. Data in (B) and (D) are presented as median ± SD of the values obtained for each time in three independent experiments. *P < 0.05; **P < 0.0003.

MCF-7 cells have been classified as poorly invasive tumor cells [22]; however, it has been previously demonstrated in other studies that under IL-1*β* stimulation they acquire migratory and invasive features; stimulated cells move towards a chemokine CXCL12 gradient and invade extracellular matrix proteins [8, 9]. To assess possible differences in chemotaxis and invasion between MCF-7pl and MCF-7A3 cells, they were stimulated with IL-1*β* for 24, 48 and 72 h prior to be seeded in Transwell migration units in serum-free culture medium and allowed to migrate for 24 h to the lower chamber of the unit containing the same medium supplemented with CXCL12. Cells that migrated through the 8 *μ*m pore filter, that separates the upper and the lower chambers, were fixed with PFA and stained with DAPI. The number of stained nuclei in each filter was counted in acquired digital images of ten randomly chosen fields in each experimental condition.  Figure 3(A) depicts the nuclei of cells that migrated at different times. Figures 3(A(a)) and 3(A(b)) show that few MCF-7pl or MCF-7A3 cells migrated in nonstimulated conditions. Prestimulation with IL-1*β* induced a gradual increase of chemotaxis (Figures 3(A(c)) and 3(A(d))) that was much higher in MCF-7A3 cells and reached a maximum value when cells had been previously exposed to IL-1*β* for 48 h (Figures 3(A(e)) and 3(A(f))). Previous exposure to IL-1*β* for 72 h, however, did not increase the number of cells that moved towards CXCL12 but decreased it considerably (Figures 3(A(g)) and 3(A(h))). The latter results could be possibly explained because cells have been in culture medium without serum for many hours or because the chemokine could have been inactivated after the long time required for the cell migration assay. Box plots in Figure 3(B) show quantification of the chemotaxis results from three independent experiments.

The invasive capacity of cells was measured with the same type of chambers, covering the filter with a layer of Matrigel. After prestimulation with IL-1*β* for 24, 48, or 72 h, MCF-7pl and MCF-7A3 cells were transferred to the upper chamber containing serum-free fresh medium without IL-1*β* and allowed to invade the protein layer for 36 h. The cells that reached the lower side of the filter were counted as indicated for the chemotaxis assays.  Figure 3(C) shows representative-acquired images of cells that invaded Matrigel at different times in which it is possible to appreciate that more MCF-7A3 cells invaded Matrigel in basal conditions than MCF-7pl cells (Figures 3(C(a)) and 3(C(b))). Invasiveness of MCF-7A3 cells previously exposed to IL-1*β* for 24 h did not further increase (Figures 3(C(c)) and 3(C(d))). The highest value was obtained when cells had been previously stimulated for 48 h (Figures 3(C(e)) and 3(C(f))). Longer times under cytokine stimulation did not increase invasiveness, but otherwise (Figures 3(C(g)) and 3(C(h))). The cause for the observed decrease could be the same already in the migration assays. Box plots in Figure 3(D) show quantification of invasiveness from three independent experiments. These results showed that MCF-7A3 cells responded more efficiently to IL-1*β* stimulus by showing increased chemotaxis to CXCL12 and increased capacity to degrade extracellular matrix proteins, particularly after being exposed for 48 h to IL-1*β*.

### 2.6. Expression of E-Cadherin mRNA and Protein in MCF-7A3 Cells

**Figure 4:**
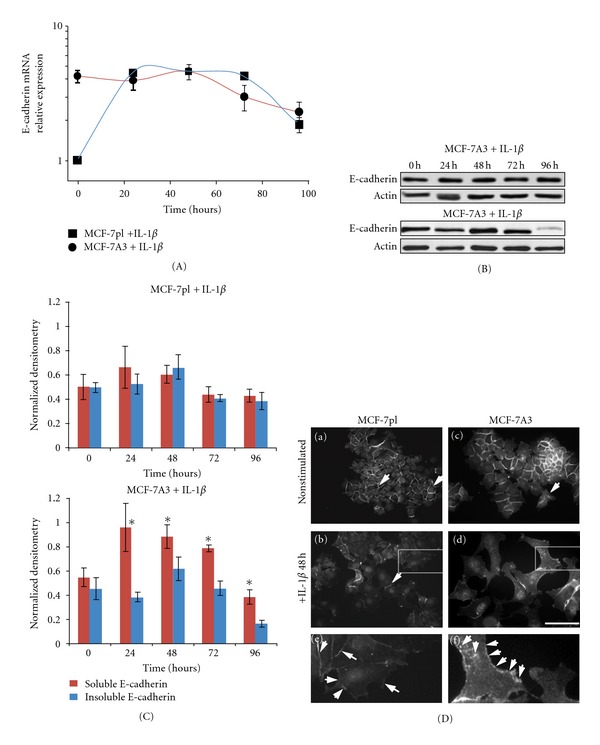
Characterization of E-cadherin mRNA expression and E-cadherin levels in MCF-7pl and MCF-7A3 cells. (A) Relative expression profile of E-cadherin mRNA. MCF7-7pl and MCF-7A3 cells were stimulated with IL-1*β* as previously indicated. After stimulus, gene expression was evaluated by qRT-PCR at the indicated time points. Data are presented as mean ± SD and normalized to the basal expression of E-cadherin mRNA in nonstimulated MCF-7pl cells (Time zero). (B) Representative Western Blots showing the levels of E-cadherin in IL-1*β*-stimulated MCF-7pl and MCF-7A3 cells at the times when E-cadherin mRNA expression was evaluated. Protein blots were challenged with anti-E-cadherin antibody. (C) Densitometric analysis of Western Blots against E-cadherin in Triton X-100 soluble and insoluble cell fractions in MCF-7pl and MCF-7A3 cells stimulated with IL-1*β* obtained from three independent experiments. Data is presented as normalized densitometry relative to time zero, where sum of densitometry values from both Triton X-100 insoluble and soluble E-cadherin bands was set equal to 1. *P < 0.05. (D) Immunolocalization of E-cadherin in MCF-7pl and MCF-7A3 cells. Nonstimulated or IL-1*β*-stimulated cells were fixed and stained for immunofluorescence with anti-E-cadherin antibody and a secondary antibody tagged with FITC and nuclei counter-stained with DAPI. Bar = 20 *μ*m. Micrographs (e) and (f) are magnifications of the cells within the rectangles in micrographs (b) and (d), respectively. Arrows indicate localization of E-cadherin.

The first changes that have been observed in nonselected MCF-7 cells stimulated with IL-1*β* were the loss of contact between adjacent cells and the delocalization of the proteins forming intercellular junctions [8, 23]. When junctions are disorganized, the proteins that form them are also disorganized, relocated, or degraded. It has been postulated that during EMT, E-cadherin is lost and substituted by N-cadherin in the cells that acquire a mesenchymal phenotype [24]. However, when this substitution takes place and to which extent E-cadherin is replaced or degraded are not yet clearly established. Recent reports have shown that the two types of cadherins can be simultaneously expressed in a cell and that N-cadherin is not present in all invasive cancerous cells [15, 25]. Moreover, there are contradictory reports about delocalization of E-cadherin from the cell membrane, its lifetime in the cytoplasm, its degradation, and its possible entry into the cell nucleus [10, 15, 25]. To analyze if the selected population of cells could be a suitable model to determine the timing and sequence of molecular changes leading to transition to an invasive phenotype, MCF-7A3 cells were employed to measure expression of E-cadherin mRNA and of protein levels before and after IL-1*β* stimulation. Quantitative RT-PCR results in Figure 4(A) show that E-cadherin mRNA expression in MCF-7pl cells was rapidly increased after stimulation with IL-1*β* and reached its highest level at 24 h. Expression remained without significant variation until 72 h, to significantly decrease at 96 h. In contrast, baseline levels of E-cadherin mRNA expression in MCF-7A3 were already 4-fold higher and were maintained up to 48 h to slowly decrease to 2-fold at 96 h.

Western Blot analysis of proteins from IL-I*β*-stimulated MCF-7pl and MCF-7A3 cell extracts did not reveal significant changes in total E-cadherin levels in MCF-7pl, whereas, in MCF-7A3 cells, a decrease was initiated before 72 h to become clearly evident at 96 h (Figure 4(B)). These experiments also show that all the protein present in the cells under the experimental conditions employed that the E-cadherin in the cells corresponded to the mature form of the protein (120 kDa), which is a product of processing of the N-glycosylated precursor (130 kDa). Loss of E-cadherin expression has been considered an important marker of cancer cell progression to malignancy [26] and several mechanisms such as activation of protooncogenes mediate phosphorylation of E-cadherin to tag it for degradation after its removal from the membrane into the cytoplasm [27]. To explore whether IL-1*β* was inducing translocation of E-cadherin to the cytoplasm, Triton X-100 soluble and insoluble subcellular fractions were prepared from IL-1*β*-stimulated MCF-7pl and MCF-7A3 cells. Soluble and insoluble cell extracts were separated, blotted, and challenged with an anti-E-cadherin antibody. Figure 4(C) shows normalized densitometric quantification of E-cadherin in subcellular fractions obtained from these cells at different times under stimulation with the cytokine. In MCF-7pl cells, the proportion of Triton-soluble E-cadherin/Triton-insoluble E-cadherin slightly increased at 24 h and decreased to a ratio close to 1 : 1 at later times. However, the difference between the two fractions was not significant. In IL-1*β*-stimulated MCF-7A3 cells, an increase close to 2-fold was registered in soluble E-cadherin that was maintained up to 48 h and then slowly decreased to very low levels at 96 h, a time in which Triton-insoluble E-cadherin also decreased drastically. The difference in the subcellular distribution of E-cadherin clearly observed in MCF-7A3 cells suggests that newly synthesized E-cadherin is found in the soluble form and it is maintained at similar levels to those of insoluble E-cadherin for at least 72 h. After this time, it is degraded together with the insoluble form, suggesting that E-cadherin mRNA expression may be regulated by the levels of delocalized E-cadherin in the cells. To further explore the cellular localization of E-cadherin, in IL-1*β*-stimulated cells, and correlate this with mRNA expression and protein levels at the different times, cells in the different conditions were fixed and stained with anti-E-cadherin antibody. A colony of MCF-7pl cells (Figurer 4 (D(a))) shows regular polygonal cells with borders in close contact with each other and E-cadherin exclusively confined to the periphery (arrows). After IL-1*β* stimulation (Figure 4(D(b)) and 4(D(e))), the protein was detected in the cytoplasm of scattered cells that had acquired a different morphology, although it was still present in the periphery of cells that remained in contact with each other (arrows). Figure 4(D(c)) shows a colony of MCF-7A3 cells where E-cadherin was localized in the periphery of only those cells that are in close contact with their neighbors, while in many cells that have detached from the colony and acquired an extended shape that occupies a larger area (arrow), E-cadherin was mostly localized in the cytoplasm. In the center of the colony, clustered cells showed E-cadherin localized at the cell periphery. Stimulation of MCF-7A3 cells with IL-1*β* induced big changes of cell morphology, and many extended cells could be seen where E-cadherin was displayed in the cytoplasm and occasionally in the membrane between cells still connected or found in small cytoplasmic particles, tethered to the cell membrane (Figures 4(D(d)), 4(D(e)), and 4(D(f))). These results showed clear correlation between the time in which degradation of E-cadherin occurred in the cells and other processes (suggestive of cell transition to an invasive phenotype) were initiated. Furthermore, they showed that precise timing of several of the phenomena induced by IL-1*β* stimulation can be determined, particularly if the changes were transient, as expected in a dynamic transition occurring in a relative short time.

## 3. Discussion

Mammary cancer is a complex disease showing different histopathological features, genetic and genomic variability, and differences in its prognosis. For this reason, there is not an individual model that recapitulates all the stages of the disease and cell lines of mammary cancer have been widely used to study to investigate cell migration, proliferation, and apoptosis; all of them are processes that become unregulated during tumor progression [28, 29]. Although many insights for the onset of cancer malignancy have been obtained using cell lines, these could undergo genotypic changes after some time in culture and often are compounded by diverse subpopulations [29–31]. Consequently, many of the currently used cell lines respond heterogeneously to a specific stimulus, making interpretation and comparison of results very difficult.

Many studies have demonstrated that, in the microenvironment of most tumors, several cellular effectors and mediators of inflammation are present [5]. Among these, high levels of the proinflammatory cytokine IL-1*β* have been related with invasion, angiogenesis and promotion of malignancy [6]. In the case of mammary cancer, high levels of IL-1*β* have also been correlated with aggressive tumors and a high recurrence in patients [32].

Recently, our group has demonstrated that the exposure of poor invasive epithelial mammary cancer MCF-7 cells to IL-1*β* induces a fibroblastoid morphology and rearrangement of actin patterns by activation of Rac1 signaling pathways; together with specific molecular changes that promoted cell scattering, migration, and invasion [8]. Additional to the molecular changes induced by IL-1*β* stimulus, increased secretion of metalloproteases and increase, expression of the chemokine CXCL12 receptor, CXCR4, were also reported [9]. In the present work, high expression of this receptor was utilized as selection marker to obtain a subpopulation of the parental MCF-7 cells that could respond in a uniform fashion to the stimulus of IL-1*β* to obtain a clear assessment of the intensity and timing of IL-1*β*-induced and/or -activated processes; which to this date remain controversial.

MCF-7 parental cells maintain expression of specific molecular markers of natural epithelial layers such as E-cadherin, claudins, and ZO-1, among other proteins that constitute the intercellular junctions. These structures are required to maintain structural integrity and the barrier function that characterize normal epithelia [22, 29]. Evaluation of the selected MCF-7A3 cell subpopulation showed that their response to IL-1*β* stimulus was significantly higher and more uniform than that of the heterogeneous MCF-7pl cells. These features were maintained at least during five culture passages. The increased response could be explained by the observed stability of the receptor IL-1RI levels on the surface of MCF-7A3 cells, as this would provide increased opportunity for binding of the cytokine and activation of signaling mechanisms.

Among the cellular processes induced by IL-1*β* in the great majority of MCF-7A3 cells are included detaching and scattering of the cells from the initially established colonies and formation of secondary or satellite colonies, all of which contained cells that were not tightly adhered to each other. The different organization between cells in these colonies bestowed MCF-7A3 cells with higher capacity to separate from their neighbors, change shape, and move away from the colony. The higher number of colonies formed by MCF-7A3 cells, together with the higher percentage of colonies containing dispersed cells after IL-1*β* stimulation, suggested a possible correlation with a higher proliferation rate. The considerable increase in number of cells being labeled with fluorescent calcein-AM that was registered as exposure to IL-1*β* progressed with time supported that proliferation rate of MCF-7A3 cells was augmented by the cytokine stimulus.

The significant increase in chemotaxis induced by IL-1*β* in MCF-7A3 cells towards chemokine CXCL12, known to be secreted by cells in target organs of invasive mammary cells and considered a marker of tumor progression to malignancy [33], could be directly correlated with the increase of CXCR4 levels and the higher stability of IL-1RI that was registered in these cells after exposure to IL-1*β*. Furthermore, higher break down of ECM proteins by MCF-7A3 cells during invasion of Matrigel layers could be explained by increased secretion of proteases MMP2 and MMP9 to the medium by the stimulated cells, as this phenomenon has been already reported to occur in the MCF-7 cells stimulated with IL-1*β* [8]. All these acquired or increased features and activities of MCF-7A3 cells stimulated with IL-1*β* were present in the great majority of them, so the results are representative of a uniform response of the selected population and not a mixture of different responses, as is the case with heterogeneous cancer cell lines. To obtain additional evidence about the advantage provided by using a selected cell population like the MCF-73A cells, these were employed to measure E-cadherin mRNA expression and the protein levels in cells stimulated with IL-1*β* for different lengths of time. E-cadherin is frequently reported delocalized from the membrane and posttranslationally modified in cancerous metastatic cells. Its deregulation during breast cancer progression has been correlated with poor prognosis [34]. Our results showed that E-cadherin baseline mRNA expression was already high in MCF-7A3 cells compared with the baseline levels in MCF-7pl cells. High expression levels were maintained after stimulus with IL-1*β* for at least 48 h, although levels of total E-cadherin did not show significant variation at this time. Distribution of the protein in subcellular fractions showed a higher proportion in Triton X-100-soluble fractions. In cells under these conditions, E-cadherin was being concentrated in the cytoplasm of scattered cells and in the membrane remaining between few connected cells, as shown in cells of Figure 4. These results suggested that E-cadherin mRNA in MCF-7A3 is stable and stimulus by IL-1*β*, that induces accumulation of soluble protein in the cytoplasm, down regulates E-cadherin mRNA expression. Once the total protein levels of E-cadherin in the cytoplasm started to decrease after 72 h, expression of its mRNA also went down and the proportion of Triton X-100-soluble protein decreased rapidly together with the insoluble protein remaining in the cells. In contrast, in MCF-7pl cells, total protein levels remained without significant variation up to 96 h and cells maintained a 1 : 1 proportion of soluble-insoluble protein, although E-cadherin mRNA levels evidently decreased after 72 h. As shown in previous reports, delocalized E-cadherin accumulated in the cytoplasm is degraded [10]. Our present results show loss of the protein, taking place in MCF-7A3 under the effect of IL-1*β* stimulus; suggesting that degradation mechanisms are activated by accumulation of E-cadherin in the cytoplasm at later times. Transition to an invasive phenotype evidently will require activation of other mechanisms, many of which are currently being investigated by several groups and beyond the scope of the present work.

The present study, which focuses on the initial phases of the transition following the sequential changes induced by IL-1*β* in a uniformly responding cell population, has corroborated the important role of IL-1*β*. We have also shown that unfeasibility to discriminate between responding and nonresponding cells in a heterogeneous cell population hinders the possibility to reach an adequate interpretation, as has been elegantly indicated and discussed by Uchino and collaborators [31]. MCF-7A3 cells showed a uniform response to IL-1*β* stimulus that allowed precise characterization of morphological, biochemical, and functional differences in cytokine-stimulated cells. It was possible to establish timing and correlations between initiation and activation of the events occurring in the cells at the onset of transition to higher invasiveness, which could not be assess in heterogeneous cell lines. Although the present approach cannot be considered a model for breast cancer, it provides an advantage to study responses and behavior of cancerous cells. In particular, the effect of a single stimulus, as is the case with the proinflammatory cytokine IL-1*β*, would have been difficult to dissect and interpret in heterogeneous populations. However, working with a selected population presents serious disadvantages that are important to keep in mind. In a tumor, in contrast to *in vitro *models, cells are not only heterogeneous, but they are immersed in diverse ecosystems and communicate with other cell compartments where many signals could participate determining the tumor phenotype and cancer progression [35].

## 4. Materials and Methods

### 4.1. Cell Culture

MCF-7 cell line was obtained from ATCC (Manasas, VA). Cells were cultured in DMEM-F12 1 : 1 (v/v) supplemented with 10% fetal bovine serum (FBS). For experiments, 6 × 10^3^ cells/cm^2^ were seeded on culture dishes and cultured for 48 h. Then, cultures were washed with PBS and switched to DMEM-F12-1% FBS for 18 h. After this period of serum starvation, cultures were switched to DMEM-F12 without serum, supplemented with 20 ng/mL of IL-1*β*, and cultured for different times in 5% CO_2_ and 37°C atmosphere. 

### 4.2. Flow Cytometry

MCF-7 cells were harvested by addition of PBS-0.5% EDTA during 30 min at 37°C. Cell suspension was washed thrice with ice-cold PBS-2% FBS and centrifuged for 5 min at 1500 rpm. Afterwards, cells were incubated on ice with PBS supplemented with 2% FBS and 16.5 *μ*g/mL human IgG (Pisa Laboratorios, Guadalajara, Mexico). Cells were challenged with one of the following antibodies: mouse anti-human CXCR4 (Santa Cruz Biotechnologies, Santa Cruz, CA), or goat anti-human IL-1RI (R&D Systems, Inc., Minneapolis, MN) for 60 min at 4°C. Secondary antibodies were FITC-labeled anti-mouse IgG and FITC-labeled anti-goat IgG (Invitrogen, Carlsbad, CA). Flow cytometry was carried out in a Becton Dickinson FACScalibur (Becton Dickinson, Mountain View, CA), for the sorting of cells a MoFlo High-performance Cell sorter was used (Beckman Coulter, Brea, CA). Data were analyzed with Summit software v5.1.

### 4.3. Cell Proliferation Assay

Cells were cultured in 96-well plates at a density of 5 × 10^3^ cells/cm^2^. After serum deprivation, cells were passed to DMEM-F12 medium supplemented with 20 ng/mL IL-1*β*. Cells were incubated for 2 h with the membrane permeable fluorochrome calcein-AM (1 *μ*M, Invitrogen) as cell proliferation marker at time zero and at 24 and 48 h after the cytokine stimulus. Fluorescence emitted by cells that internalized and processed calcein-AM was measured in a Biotek Synergy HT fluorometer (Winoosky, VT) at 485 nm wavelength for excitation and 520 nm for emission. Calcein-AM-derived fluorescence was related to the number of live cells present in each well interpolating the values in a standard curve.

### 4.4. Chemotaxis and Invasion Assays

Cultures of MCF-7 cell after 24, 48, and 72 h from IL-1*β* stimulus were harvested with PBS-0.05% EDTA, and 105 cells were seeded in the inner chamber of Transwell units (Corning Mexicana, San Nicolas, Mexico) filled up with DMEM-F12 free of serum. The outer chamber of the Transwell unit was loaded with serum-free DMEM-F12 supplemented with 100 ng/mL CXCL12 as the chemoattractant. Cells were allowed to migrate for 24 h at 37°C. The porous membranes containing cells that migrated through it were cut out from the inner chambers and fixed with 3.4% paraformaldehyde (PFA) for 30 min. Fixed cells were permeabilized with PBS-0.2% Triton X-100 and stained with DAPI. Membranes were mounted on Vectashield (Vector Laboratories, Burlingame, CA). The rate of cell migration in each condition was evaluated by counting fluorescent nuclei in 10 fields randomly chosen in the membrane. Invasiveness of MCF-7 cells was measured in a similar way but using Matrigel-covered polycarbonate filters as a substrate for degradation by the invading cells and using serum-free DMEM-F12 medium without CXCL12 in the lower chamber and letting the cells move across Matrigel for 36 h.

### 4.5. Quantitative Gene Expression Analysis

Total RNA was extracted from cell cultures with Trizol (Invitrogen; Carlsbad, CA). Total RNA was converted to cDNA by using SuperScript II kit (Invitrogen). Quantitative RT-PCR was performed using FastStart SYBR Green Master (Rox) (Roche Applied Science; Manheim, Germany) in a Real Time 7000 Thermal Cycler (Applied Biosystems; Carlsbad, CA). Specific oligonucleotides for E-cadherin and housekeeping rplp0 genes are listed in Supplementary Material (see Table  S1 available online at doi:10.1155/2012/609148) Relative expression levels were calculated by using 2^−ΔΔCt^ equation. 

### 4.6. SDS-PAGE and Western Blot

Protein extracts were obtained lysing cell with a solution containing 50 mM Tris-HCl pH, 7.5, 5 mM EDTA, 150 mM NaCl, 1% Triton X-100, protease inhibitor cocktail (Roche Applied Science Manheim, Germany), 1 mM sodium fluoride, and 1 mM sodium orthovanadate. Protein extracts were sonicated employing 3 pulses of 10 s at 40% amplitude, and, after centrifugation at 12,000 rpm for 3 min, the supernatant was recovered. Thirty micrograms of protein were loaded per lane, separated in 10% SDS-PAGE, and blotted onto nitrocellulose membranes. After blocking with TBS-0.1% Tween 20–6% BSA membranes were challenged with anti-E-cadherin antibody (Santa Cruz Biotechnologies). As loading control, anti-*β*-actin antibody (kindly provided by Dr. J. M. Hernández; CINVESTAV-IPN, Mexico) was utilized. HRP-conjugated secondary antibodies were all from Sigma (Saint Louis, MO). Positive bands were revealed by enhanced chemiluminescence. Triton X-100 insoluble and soluble cellular fractions were prepared as previously reported [36] and separated, blotted and challenged with anti-E-cadherin antibody as done with the total protein extracts.

### 4.7. Immunofluorescence

All the cells were prepared for immunofluorescence assays as has been described for MCF-7 cells in previous reports from our group [8, 9]. The antibody to E-cadherin was the same as the one used for Western Blots, but utilized at 1 : 50 dilution. The secondary antibody was an anti-mouse IgG tagged with FITC used at 1 : 200 dilution. Images were taken with an Olympus IX 50 inverted fluorescence microscope. All images were acquired employing the LC Plan FL 40X objective and the software Image-Pro Plus (v.5.0) from Media Cybernetics (Silver Spring, MD).

### 4.8. Statistical Analysis

Mann-Whitney test for the comparison of two data groups and Kruskal-Wallis test for three of more data groups were chosen to analyze statistical differences. Data are presented as mean plus/minus standard deviation or median and range as indicated. Statistical differences were set with a *P* value equal or lower than 0.05. Each result represents at least three independent experiments.

## Supplementary Material

List of the oligonucleotides used as primers for the quantification of the expression of E-Cadherin, using *rplpO* housekeeping gene as normalizing factor. Quantitative RT-PCR was monitored by Sybr Green I fluorescent dye and fold change was determined by 2'^AACt^ formula.
