# Supplementary material for: Selection of a MCF-7 Breast Cancer Cell Subpopulation with High Sensitivity to IL-1β: Characterization of and Correlation between Morphological and Molecular Changes Leading to Increased Invasiveness
Source: Int J Breast Cancer. 2012 May 10;2012:609148. doi: 10.1155/2012/609148 (PMC3357940; doi:10.1155/2012/609148)
Supplement: Supplementary file 1 — List of the oligonucleotides used as primers for the quantification of the expression of E-Cadherin, using rplpO housekeeping gene as normalizing factor. Quantitative RT-PCR was monitored by Sybr Green I fluorescent dye and fold change was determined by 2'AACt formula. [file 609148.f1.doc]

| Gene | Genbank Accesion Number | Sequence | Ta |
| --- | --- | --- | --- |
| E-Cadherin (*cdh-1*) | NM_004360 | 5’-CCCACCACGTACAAGGGTC-3’ | 60 ºC |
|  |  | 5’-CTGGGGTATTGGGGGCATC-3’ |  |
| Ribosomal Protein Large P0 (*rplp0*) | NM_ 001002 | 5’-ATGGGGAAGCTGAAGGTCGG-3’ | 60 ºC |
|  |  | 5’-GTGGCAGTGATGGCATGGACT-3’ |  |
